# An Open Source Image Processing Method to Quantitatively Assess Tissue Growth after Non-Invasive Magnetic Resonance Imaging in Human Bone Marrow Stromal Cell Seeded 3D Polymeric Scaffolds

**DOI:** 10.1371/journal.pone.0115000

**Published:** 2014-12-12

**Authors:** Anne M. Leferink, Raluca M. Fratila, Maaike A. Koenrades, Clemens A. van Blitterswijk, Aldrik Velders, Lorenzo Moroni

**Affiliations:** 1 Department of Tissue Regeneration, MIRA – Institute for Biomedical Technology and Technical Medicine, University of Twente, Enschede, The Netherlands; 2 NeuroImaging Group, MIRA – Institute for Biomedical Technology and Technical Medicine, University of Twente, Enschede, The Netherlands; 3 Department of Complex Tissue Regeneration, Faculty of Health, Medicine and Life Sciences, Maastricht University, Maastricht, The Netherlands; 4 Laboratory of SupraMolecular Chemistry and Technology of MESA – Institute for Nanotechnology, University of Twente, Enschede, The Netherlands; 5 Department of BioNanotechnology, Agrotechnology and Food Sciences, Wageningen University, Wageningen, The Netherlands; Michigan Technological University, United States of America

## Abstract

Monitoring extracellular matrix (ECM) components is one of the key methods used to determine tissue quality in three-dimensional (3D) scaffolds for regenerative medicine and clinical purposes. This is even more important when multipotent human bone marrow stromal cells (hMSCs) are used, as it could offer a method to understand in real time the dynamics of stromal cell differentiation and eventually steer it into the desired lineage. Magnetic Resonance Imaging (MRI) is a promising tool to overcome the challenge of a limited transparency in opaque 3D scaffolds. Technical limitations of MRI involve non-uniform background intensity leading to fluctuating background signals and therewith complicating quantifications on the retrieved images. We present a post-imaging processing sequence that is able to correct for this non-uniform background intensity. To test the processing sequence we investigated the use of MRI for *in vitro* monitoring of tissue growth in three-dimensional poly(ethylene oxide terephthalate)–poly(butylene terephthalate) (PEOT/PBT) scaffolds. Results showed that MRI, without the need to use contrast agents, is a promising non-invasive tool to quantitatively monitor ECM production and cell distribution during *in vitro* culture in 3D porous tissue engineered constructs.

## Introduction

A typical tissue engineering approach consists of combining cells with a synthetic or biological porous material called scaffold, which provides a mechanically stable environment to culture a substitute graft *in vitro* before implantation. Prior to implantation, several parameters including cellular distribution, extra cellular matrix (ECM) formation and tissue functionality need to be assessed. Monitoring in real-time these parameters is even more relevant when multipotent bone marrow stromal cells are used, as it is known that these cells can eventually progress into a non-desired cell phenotype when cultures on three-dimensional (3D) scaffolds are not controlled. For example, Jukes *et al.* and Scotti *et al.* showed that both embryonic and mesenchymal stem cells differentiate into a mature osteogenic lineage by first passing by chondrogenesis [1], [2]. Therefore, monitoring tissue growth and development through analyzing the ECM that is formed in time would be a valuable tool to step from a conventional approach where scaffolds are treated as a “black-box” during culture to a new phase where the information obtained through monitoring could eventually be utilized to steer cell differentiation into a targeted phenotype [3], [4].

Unfortunately, 3D scaffolds for tissue engineering strategies are often composed of polymeric materials with limited transparency. This property restricts the applicability of several imaging techniques like confocal microscopy, Raman spectroscopy and second harmonic generation imaging to superficial imaging of the constructs [5]. Histology is the most commonly used and most informative method to identify cells and ECM components inside scaffolds. However, this destructive method does not allow for real-time monitoring of tissue growth on a single sample. To be able to monitor cell growth and ECM formation over time by histological analysis, multiple samples have to be processed. Moreover, the obtained sections only represent part of the scaffold, which complicates the determination of cell growth and ECM formation for the whole construct. Micro-computed tomography (µ-CT) can overcome these problems of limited transparency and sample preparation of a tissue engineered construct, but uses ionizing radiation and requires dense tissue to provide contrast [6]. Positron Emission Tomography (PET) and Single Photon Emission Computed Tomography (SPECT), generally combined with computed tomography (CT), can be applied to localize and study tissue dynamics like tissue metabolism. However, these imaging modalities require the use of radioactive tracers [7].

A promising approach to monitor cellular distribution and tissue formation in time in 3D is to use magnetic resonance imaging (MRI), also referred to as magnetic resonance microscopy (MRM) when applied with very high resolution, which is a non-invasive, non-ionizing and optionally label-free tool [8], [9]. MRI has the capability to image thin slices of tissue with a sub-millimeter resolution in any orientation at any depth. Contrast in label-free MRI images is based, among others, on variations in tissue hydration or water/lipid ratios, which results in differences in the spin-phase and relaxation time of protons. For example, in previous work on magnetic resonance (MR) detection in bone tissue engineering approaches, an inverse relationship between MR relaxation times and mineral concentration was found after culturing osteoblasts on poly-(ethyl methacrylate) (PEMA) scaffolds [5]. A correlation between collagen orientation and water proton transverse relaxation times (T_2_) was found in articular cartilage [10], [11] and in tendon under load [12]. In several other studies contrast agents were implemented in MRI to track cells both *in vitro* and *in vivo* [13]. Immobilization of contrast agents on nanoparticles to permit endocytosis is required for cell labeling [14], [15]. After endocytosis of the contrast agent, depending on the type of contrast agent, accumulation of the agent leads to a darker or brighter signal, which will either reduce or increase the contrast between the labeled cells and the scaffolds [16]. A hurdle that has to be overcome is the possible loss of signal over time and/or increase in false positive signal both due to agent clearance or agent diffusion. Another challenge to take into account is that by incorporating MRI contrast agents to assess the quality of the tissue construct, the application of the labeled construct in the clinics might be complicated by regulations with respect to the exact formulation of the construct. Although contrast agents are already widely applied in clinical practice, their use is restricted to specific diagnostic procedures and limited to non-toxic doses.

Label-free micro-MRI has already been applied in tissue engineering approaches where dense tissues were assessed to detect for example collagen orientation and collagen mineralization [11], [17]. However, the method of micro-MRI still has some challenges with respect to contrast with low tissue density, resolution, and background intensity inhomogeneities. In this study, we present a freely accessible novel image processing method to overcome the limitation of inhomogeneous background intensity in MRI data. Subsequently to image processing, quantitative information in 3D by image analysis was retrieved without the need to use contrast enhancing agents during scanning. To test our image processing method, we have cultured MSCs in 3D rapid prototyped scaffolds as a model 3D cell culture system and quantified the tissue growth and distribution throughout these scaffolds after MRI. We show that by application of our image processing method we were able to detect very small amounts of tissue which normally would not be detected by unprocessed MRI. In unprocessed images the background intensity fluctuations are found to be dominant over the contrast between the low density tissue and PBS within the pores of the scaffold.

With this method, small amounts of tissue and its distribution were monitored for the first time non-destructively within a clinically relevant sized opaque 3D construct cultured *in vitro* for tissue engineering applications.

## Materials and Methods

### Isolation of human bone marrow derived stromal cells

Bone marrow aspirates were obtained upon approval of the Medical Ethical committee of the local hospital (Dutch: Medisch Ethische Toetsingscommissie (MECT) van het Medisch Spectrum Twente) following the Dutch national ethics guidelines from patients who had given written informed consent. Human MSCs from two donors (donor 1: female, age 77; donor 2: female, age 55) were isolated in approval to the local national ethical committee and expanded as described previously [18]. Briefly, aspirates were resuspended using a 20-gauge needle, plated at a density of 50,000 cells/cm^2^ and cultured in proliferation medium, which contains minimal essential medium (alpha-MEM; Life Technologies, Gaithersburg, MD), 10% heat-inactivated fetal bovine serum (FBS; Lonza), 0.2 mM L-Ascorbic acid 2-phosphate magnesium salt (ASAP, Sigma Aldrich), 2 mM L-glutamine (L—glut, Invitrogen), 100 units/mL penicillin (Life Technologies), 10 µg/mL streptomycin (Life Technologies) and 1 ng/mL basic fibroblast growth factor (bFGF; Instruchemie, Delfzijl, The Netherlands). Cells were grown at 37°C in a humidified atmosphere with 5% CO_2_. Medium was refreshed twice per week and cells were used for further subculturing or cryopreservation on reaching near confluence.

### Fabrication of PEOT/PBT scaffolds

Scaffolds were fabricated of 300PEOT55PBT45 (PolyActive300/55/45, PolyVation, The Netherlands) which is a block copolymer with a weight ratio of 55 to 45 for the two PEOT and PBT components respectively, and a molecular weight of 300 Da for the PEG segments used in the co-polymerization process. PEOT/PBT copolymers have been extensively studied and proved to be biocompatible *in vitro* as well as *in vivo* [19] [20]. Therefore, the material is introduced here as a model material to fabricate biocompatible 3D cell culture scaffolds. Cylindrical porous scaffolds (8 mm in diameter by 3 mm in height) were fabricated as described before [21] by fused deposition modeling with a bioscaffolder (SysENG, Germany) with a fiber to fiber distance of 1000 µm, a fiber diameter of approximately 200 µm, and a layer thickness of 150 µm. These scaffolds were sterilized in 70% ethanol 2 times for 30 minutes each, washed in PBS first for 5 minutes and additionally for other 30 minutes two times, and finally incubated in culture medium overnight prior to cell culture.

### Cell seeding of PEOT/PBT scaffolds

Scaffolds were dried and transferred to non-treated 24-well plates (NUNC). The hMSCs (passage 3) were harvested from monolayer expansion and suspended in proliferation medium. The scaffolds were seeded with 750,000 hMSCs in 100 µL of medium which was previously shown as an optimal cell density for this scaffold volume to reduce cell loss upon seeding [22]. The cell suspension was gently resuspended on top of the scaffolds to fill all the pores of the scaffold. After 1.5 hour of incubation in the given volume, the medium was filled up to 500 µL. Proliferation medium was replaced twice a week for all samples. Constructs were cultured up to 60 days.

### MRI equipment and measurements

MRI experiments were performed at room temperature on a 14.1 T (600 MHz) Avance II NMR spectrometer from Bruker (Karlsruhe, Germany), equipped with a vertical narrow bore magnet, a Great B0 compensation unit, and 3 Great 1/60 amplifier units (X, Y, and Z). A micro 5 imaging probe with a 10 mm diameter saddle coil insert from Bruker (Karlsruhe, Germany) was used. Images were obtained using a multi-slice-multi-echo (MSME) sequence with the following parameters: repetition time (TR)  = 1000 ms, echo time (TE)  = 10 ms, flip angle  = 90°, slice thickness  = 0.07 to 0.1 mm, inter-slice gap  = 0, field of view (FOV)  = 1 cm, and 128×128 or 256×256 matrix. To optimize scanning parameters the first samples were imaged in phosphate buffered saline (PBS) after 30 minutes of fixation in 10% formalin. Subsequently, a bare scaffold was immersed in PBS and scanned after degassing as a non-tissue control. This bare scaffold was then incubated in medium prior to cell culture. Additionally, the optimized scanning parameters were applied on this previously scanned scaffold and on other unfixed samples residing in PBS during scanning. To investigate if non-targeted contrast agents can increase the contrast between the solution in which the samples were scanned and the lipids present in the tissue of the sample, one scaffold was imaged before and after addition of Endorem (Guerbet, Villepinte, France). The Endorem solution is comprising superparamagnetic iron oxide nanoparticles (11.2 mg/ml) dispersed in water and was added to PBS with a concentration of approximately 0.5% (v/v). The particles are composed of several iron oxide cores (diameter 4–6 nm) embedded inside a dextran coating resulting in an estimated hydrodynamic size of 80–150 nm [23], [24]. The Endorem T_2_ contrast agent was added to a fixated sample to change the magnetic resonance properties of the solution without inducing cellular uptake.

To investigate whether the tissue constructs can be kept in culture after a non-invasive MRI measurement, which is a requirement for longitudinal studies, un-fixated samples were carefully transferred to a sterile MRI tube and imaged in culture media. Subsequently, samples were placed back in the culture plate and culture was continued.

### Image processing and quantification

MRI images were processed in Fiji [25] prior to analysis to correct for non-uniform background intensity and to reduce noise. Background approximations were extracted from the top or bottom slice, where no scaffold and tissue material were present, by performing morphological opening with a disk shaped structuring element (radius 9, Gray Morphology plugin). Background approximations were subtracted from the original image stack to remove the non-uniform intensity. A median filter (pixel radius 2) was applied to the image stacks for noise reduction. After processing, only tissue structures appeared as bright regions, while water and scaffold material showed distinguishably lower intensities, enabling unique identification of constituents of interest. Images were segmented based on a single intensity threshold within a circular region of interest (ROI), comprising scaffold and surrounding water within the MRI glass tubes [26]. Subsequently, morphological opening (pixel radius 2) was applied to smooth the objects and remove outliers. Due to changing overall brightness and intensities between different experiments, the threshold had to be set for each experiment individually. To identify tissue material the threshold was set as such that a bare scaffold, without the presence of cells, showed a limited amount of false positive signal. The retrieved binary images were both qualitatively and quantitatively analyzed. The area of the identified objects was computed in a section-by-section (top, middle, bottom) manner to provide insight in the volumetric densities throughout the scaffolds. Per section, the binary images were merged into one 2D projection as a 4-stage look-up table. In this table colors are applied as an indication of the number of slices within the stack, in which each specific pixel in 2D projection was identified as tissue.

To quantify the amount of tissue per slice, each slice was converted into a binary image by applying a low threshold-value to identify the pixels representing scaffold material. Subsequently, these pixels identified as scaffold were subtracted from the total amount of pixels within the ROI in that respective slice. Herewith, an indication of available pore volume was retrieved. The amount of tissue was calculated as a percentage of the pixels presenting the available pore volume occupied by pixels identified as tissue.

### 3D visualization

Triangulated 3D surface meshes were created from the binary images for both scaffold and tissue material, using the integrated marching cubes algorithm in the BoneJ plugin (resampling factor 1 and 2 respectively) [27], [28]. The mesh models were imported into MeshLab (v.1.3.2), an open source tool developed with the support of the 3D-CoForm project, to render 3D models comprising tissue material and scaffold. A non-shrinking Taubin filter was applied to smooth the objects [29]. Surfaces were flat-shaded to enhance the faceting effect.

### Methylene Blue staining

To localize cells in the 3D scaffold prior to imaging with MRI, samples were washed gently with PBS, fixated in 10% formalin for 30 minutes and subsequently stained for 60 seconds using a 1% methylene blue solution in 0.1 M borax buffer (pH = 8.5, Sigma). Scaffolds were subsequently washed with demineralized (DI) water until the water was clear. The scaffolds were imaged with a Nikon SMZ800 Stereomicroscope equipped with a QImaging Retiga 1300 camera.

### Scanning Electron Microscopy (SEM)

Cell morphology and attachment was characterized by SEM analysis with a Philips XL 30 ESEM-FEG. Samples were fixated for 30 minutes in 10% formalin. Subsequently the samples were dehydrated in sequential ethanol series and critical point dried from liquid carbon dioxide using a Balzers CPD 030 Critical Point Dryer. The constructs were gold sputter coated (Cressington) prior to SEM analysis.

### Histological analysis

After MRI measurements, samples were dehydrated using a sequential ethanol series (60, 70, 80, 90, 96 and 100% ethanol, 30 minutes for each step), and subsequently embedded in glycol methacrylate (GMA). The obtained blocks were sectioned at 5 µm intervals, and stained with hematoxylin and eosin (H&E, Sigma) for visualization of the nuclei and cytoplasm.

### Statistical analysis

Quantitative results are presented as mean ± standard deviation and compared using one-way ANOVA (multiple conditions) with a Bonferroni post-test. Statistical significance was set to p- value <0.05 (*); <0.01 (**) and <0.001 (***).

## Results

### Detection of tissue on planar MRI images

**Figure 1:**
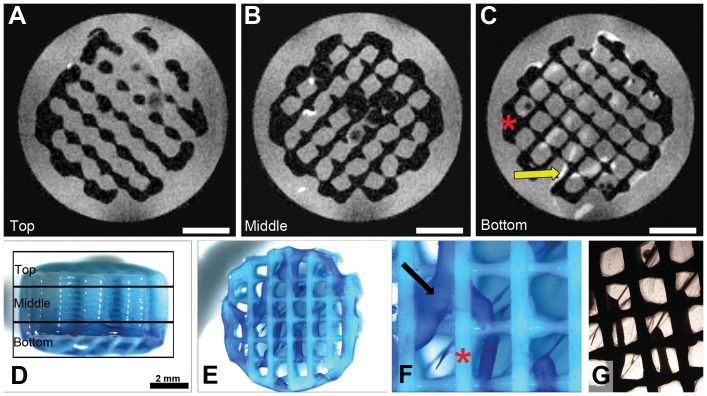
MRI scans of a scaffold after 14 days of culture with hMSCs (donor 1) taken with slice thicknesses of 80 µm. Slices from one scan from the top (A), middle (B) and bottom (C) of the scaffold areas are represented, respectively, as indicated in the marques in (D) the side-view of the scaffold in which cells are stained with methylene blue. (E-F) From the bottom-view of the scaffold, it can be seen that methylene blue stained hMSCs and ECM formed with the appearance of a dense string-like tissue (indicated with arrows) distributed non-homogeneously throughout the scaffold (indicated with asterisks). (G) This was also observed during culture by bright field microscopy. Scale bars represent 2 mm.

MRI measurements were successfully performed on a PEOT/PBT scaffold seeded with hMSCs after 14 days of static culture (**Fig. 1A-C**). A montage of all slices in this measurement can be found in S1 Figure. Prior to imaging the cells were fixated with 10% formalin and stained with methylene blue to localize tissue and cells (Fig. 1D-F). The MRI images showed a high contrast between the scaffold material (black), water (grey) and tissue-like material containing lipids (white) (Fig. 1C). As confirmed by methylene blue staining, the majority of the cells were found in the bottom layers of the scaffold. From the bottom-view of the scaffold shown in Fig. 1E and 1F similar string-like tissue patterns were found as in the MR images of the bottom slices (Fig. 1C). These string-like patterns were observed before in studies with hMSCs on the same type of scaffolds and could be a result of aggregating cells [22]. Also, bright field microscopy observations during culture prior to fixation in this study revealed this same inhomogeneous tissue distribution (Fig. 1G).

### Development of image processing sequence and 3D visualization

**Figure 2:**
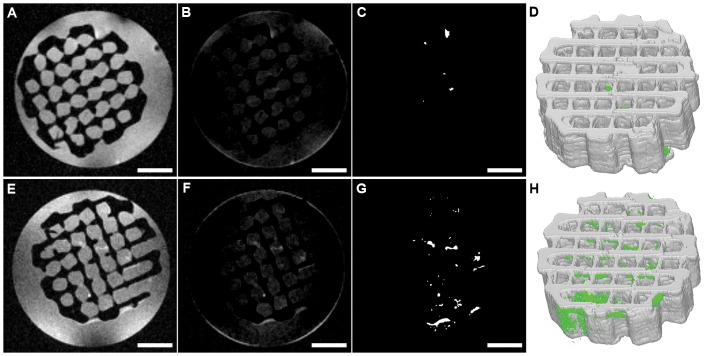
Image processing steps on MRI-derived image stacks in the absence (top, images A-D) and presence (bottom, images E-H) of hMSCs (donor 1, 5 weeks of culture). A and E represent the unprocessed MR images; B and F the images after background subtraction; C and G show the results of segmentation based on a single intensity threshold within a circular ROI, comprising scaffold and surrounding water within the MRI glass tubes; D and H are the obtained 3D mesh models, representing the tissue localization, amounts and density. Scale bars represent 2 mm.

The results of the optimization of the major processing steps and the retrieved 3D models are shown in **Fig. 2**. The complete stack of unprocessed images can be found in the supplementary information in S2 and S3 Figures. Fig. 2 A-D show the results of image analysis after MRI on a bare scaffold and Fig. 2 E-H show the results of the same scaffold after 5 weeks of static culture. In Fig. 2A and 2E the unprocessed image data as retrieved in TIF-format from the MRI software are presented. In Fig. 2B and 2F the resulting images after background subtraction are shown. Although, the intensity values of the background still showed area related variances, these variances did not seem to influence the segmentation process (Fig. 2C and 2G), since the background pixel intensity was sufficiently lower than the threshold value applied for segmentation. When comparing the two 3D models without and with hMSCs after 5 weeks of static culture (Fig. 2D and 2H respectively) tissue was clearly detected in the scaffold cultured with hMSCs. Only a minor amount of false positive signal was observed in the bare scaffold after applying the determined threshold value. Therefore, we consider the method for threshold value determination appropriate for further tissue detection in additional scans.

### The effect of Endorem contrast agent on the reliability of the 3D models

**Figure 3:**
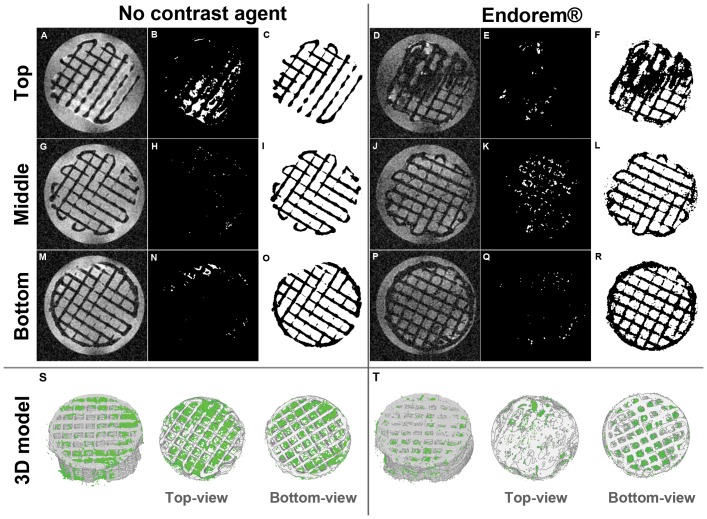
MRI scans presented by a single slice from each of the following volumes; top, middle and bottom of the scaffold after 5 weeks of culture (donor 2) obtained without the use of any contrast agents (A, G and M) and after the addition of Endorem T_2_ contrast agent (D, J and P). Tissue is identified and appears white after image processing (B, H, N, E, K and Q). Scaffold material is extracted from unprocessed images by image processing sequences (C, I, O, F, L and R). By a mathematical combination of the images representing tissue-material and the images displaying scaffold material, a 3D model could be retrieved (S and T).

To investigate whether the use of an untargeted contrast agent would increase the contrast between the PBS, the scaffold material and lipids in the tissue, two scans were performed subsequently on one scaffold which was statically cultured with hMSCs (donor 2) for 5 weeks. **Fig. 3** shows the results from a single slice per volume at different heights in the scaffold. The full stack of processed slices, representing the complete scaffold volume, can be found in S4-S7 Figures. The contrast between tissue and PBS was increased after the addition of Endorem, while the contrast between PBS and scaffold material was reduced. This resulted in an inaccurate segmentation of the scaffold (Fig. 3F, L and R), leading to closure of the scaffold pores upon 3D modelling, which can be seen in the top-view (Fig. 3F and T).

### Quantification of tissue distribution throughout the 3D scaffold

**Figure 4:**
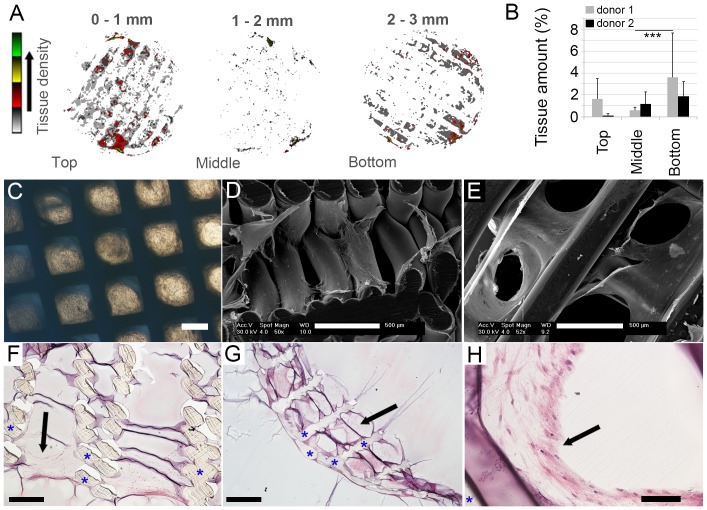
Processed images can be analysed to quantify tissue formation with high spatial resolution throughout the 3D construct. (A) Z-stacks were retrieved by summarizing multiple slices into one projection as a 4 stage look-up table. The colors represent the amount of tissue in x-y location over the full thickness of the projection as indicated by the legend. (B) By determination of the amount of tissue over a subset of slices, an indication of tissue presence in different areas of two scaffolds after 5 weeks of culture can be given (n = 15 and 18 slices per presented volume for donor 1 and donor 2, respectively). The tissue amount is presented as the percentage of the available pore volume which is occupied by tissue. The error bars represent the standard deviation in tissue amount over the different slices per scaffold volume (***: p<0.001). (C) Bright field microscopy observation through the full scaffold height in the z-direction showed a homogeneous distribution of cells and tissue between the pores in the x-y plane. By varying the focal plane upon observation an impression of pore filling per scaffold part (bottom, middle, top) can be retrieved. (D) By SEM analysis on a top-to-bottom cross-section of the scaffold, tissue is observed throughout the scaffold. Yet, from the bottom-view was observed that large, more dense, cell sheets were found on the bottom of the scaffold. (F) This finding was confirmed by histological analysis of a top-to-bottom cross-section of a construct. (G, H) From a section of the bottom layer of the scaffold it can be observed that the tissue forms circular patterns in the pores of the scaffolds. Arrows indicate cells and tissue, asterisks indicate scaffold material. Scale bars represent (C, D, E and F) 500 µm, (G) 1 mm and (H) 100 µm.

After image processing the retrieved binary images can be used for quantification and visualisation of tissue amounts. Fig. 4A shows merged projection of stacked binary images per scaffold section. By determination of the percentage of available pore volume which was found occupied by tissue per slice, (**Fig. 4A**) information was retrieved on the amount of tissue in the bottom, middle and top of the scaffold as presented in Fig. 4B (n = 15 or 18 slices per presented volume for donor 1 and donor 2, respectively). These results were confirmed by bright field microscopy observation (focal plane set on the bottom of the scaffold) and SEM analysis in which a cell sheet is found at the top and at the bottom of the scaffold (Fig. 4C and 4E), while in a cross-sectional view lower numbers of cells were found in the interior of the scaffolds (Fig. 4D). This observation is further confirmed by histological analysis (Fig. 4F: cross-section in Z-direction; Fig. 4H: a section of the bottom plane of the scaffold).

## Discussion

Currently, many studies focus on monitoring hMSCs fate in engineered tissues 3D constructs. Most conventional methods to study cell growth and distribution are based on destructive methods such as histological analysis and biochemical assays. Non-invasive imaging modalities are promising tools to non-destructively and ultimately longitudinally or continuously monitor tissue development in 3D scaffolds [9], [30]. Magnetic resonance modalities have gained interest in the field of tissue engineering for several years. First, nuclear magnetic resonance spectroscopy (NMR) has been applied to monitor metabolites in tissue engineered constructs [31]. More recently, MRI has been applied because of the capability to distinguish between several tissue types and densities such as collagens, mineralized tissues and soft tissues [8], [12], [17], [30], [32]–[34]. Contrast with a sub-mm scale resolution in MRI is obtained by variations in proton dynamics which results in different water proton transverse (T_2_) relaxation times and magnetization transfer ratios [10]. Burg *et al.* have observed that the signal intensity in MRI on 3D scaffolds was higher in cells than in the liquid phase and scaffold material [35]. The obtained contrast allowed them to observe cellular distribution in formalin fixated tissue constructs. Abarrategi *et al*. have shown to successfully assess the distribution of MSCs in 3D rapid prototyped polymeric and ceramic scaffolds without formalin fixation [30]. However, the seeding density of the constructs was relatively high with 5×10^6^ cells/scaffold on a cylindrical scaffold with a diameter of 4.5 mm and a height of 3 mm. We observed that lower densities of cells, as applied in our study (7.5×10^5^ cells) in a cylindrical scaffold with a diameter of 8 mm and a height of 3 mm, results in limited contrast. Additionally, the more homogenous a certain number of cells are distributed, the lower the contrast in MRI, probably because a lower tissue density results in a higher PBS content between cells. As a consequence, non-uniformity of intensity, which we also observed in the work of Abarrategi, further complicates quantification of tissue development, since the contrast between PBS and lipids becomes smaller than the variations in the background signal [36], [37].

To overcome this technical challenge, we presented a sequence of freely accessible image post-imaging processing steps to be able to quantify tissue growth in low density tissue constructs. Our method allowed for the identification of tissue formation in opaque mm-sized 3D scaffolds from MRI-derived image stacks. In contrast to studies that require contrast agents to localize cells, we showed that more faithful 3D models of cell and tissue distribution within solid scaffolds were obtained without the use of contrast agents. This might be due to the fact that the addition of contrast agent resulted in false positive identification of scaffold material as shown in Fig. 3T.

The single slice images in Fig. 3B and 3N could give the impression that more tissue was identified in the top volume of the scaffold than in the bottom volume. However, quantification of the tissue amounts, as presented in Fig. 4B, shows higher volumes in the bottom volume of the scaffold. This discrepancy can be caused by the fact that Fig. 3B and 3N represent only a single slice out of a stack of slices that are taken from a specific scaffold volume, whereas Fig. 4B shows the results of each stack consisting of all 15 or 18 slices in a scaffold volume. Furthermore, the tissue amount in Fig. 4B is presented as the percentage of available pore volume which is covered by tissue.

Several studies have reported on MRI analysis *in vitro* after fixation of the samples in formalin [30], [38], [39]. Studies of Fishbein *et al*. and Zheng *et al*. showed that phosphate salts and fixation solutions have an influence on the relaxation times of water protons, attributed to the physicochemical interactions (e.g. noncovalent binding or chemical exchange) of free water with polyoxymethylene oligomers formed when formaldehyde is dissolved in water. Changes in proton dynamics in these studies were found in native cartilage tissues, in which the fixatives also showed to influence the composition of cartilage over time. In our study, both un-fixated samples and fixated samples were imaged, yet no evidence was found in the unprocessed images for differences in contrast or signal intensity due to the fixative. The variations in background intensity within a slice and between different slices within one scan were dominant over any possible intensity change caused by the application of fixatives. We therefore concluded that the influence of fixatives on proton dynamics did not interfere with the post-imaging processing method to identify and quantify tissue formation. To label-free monitor tissue growth longitudinally in 3D scaffolds, without interrupting the culture phase, we believe that MRI will be a valuable tool. MRI will allow the maintenance of sterile culture conditions during scanning by controlling temperature and gas exchange, without compromising the quality of the images [40]. Furthermore, targeted contrast agents such as antibody functionalized iron oxide magnetic beads could be developed in future studies to be able to characterize the type of tissue formed by for example distinguishing between collagen-type-1 and collagen-type-2 in the ECM. Currently, MRI analysis suffer from previously mentioned technical limitations which are compromised by longer scanning times to be able to average over more multiple signals per voxel. By applying our optimized image processing sequences, based on already available image processing tools, the required duration of scanning could be reduced. We showed that our method allows us to identify very low amounts of non-mineralized tissue (1–4 volume %) in opaque polymeric scaffolds, which to the best of our knowledge had not been shown before by MRI for tissue engineering applications.

## Conclusion

The free-of-charge post-imaging processing sequence presented in this study can be a valuable tool to overcome technical limitations of image analysis after MRI. We have shown that our method enables the identification and quantitative localization of tissue in 3D opaque constructs for tissue engineering approaches. Identified tissue distribution was quantified from the image stacks, visualized in 3D mesh models and confirmed qualitatively by histological analysis. In the future, optimized post-image processing sequences can help to access shorter scanning times and improved resolution of tissue engineered constructs prior to implantation.

## Supporting Information

S1 Figure **S1 Figure represents the unprocessed images of a scan presented in** **Fig. 1** **in the main text.** The scan was performed after 14 days of culture. The last few slides of the stack show higher amounts of white areas which could represent tissue-like material.
(TIF)

S2 Figure **S2 Figure represents the unprocessed images of a scan presented in** **Fig. 2** **in the main text.** The scan was performed on a bare scaffold without any cells. There are no evident white areas with similar patterns as found on scans of scaffolds cultured with cells. The scan of the bare scaffold shows strong contrast between the scaffold material and the surrounding PBS.
(TIF)

S3 Figure **S3 Figure represents the unprocessed images of a scan performed on the same scaffold as S2 Figure yet after 5 weeks of culture.** There are small white lines visible in the first few slices of the stack. These white lines show similar patterns as found on other scans of scaffolds cultured with cells.
(TIF)

S4 Figure **S4 Figure represents identified tissue-like material (black pixels) after processing of images retrieved without the application of contrast agents.** These masked binary images are applied to establish 3D models from the full stack as presented in Fig. 3 in the main text.
(TIF)

S5 Figure **S5 Figure represents identified scaffold material (white pixels) after processing of images retrieved without the application of contrast agents.** These masked binary images are applied in combination with the images with identified tissue (S4 Figure) to establish 3D models from the full stack as presented in Fig. 3 in the main text.
(TIF)

S6 Figure **S6 Figure represents identified tissue-like material (black pixels) after processing of images retrieved after addition of a contrast agent to the PBS.** These masked binary images are applied to establish 3D models from the full stack as presented in Fig. 3 in the main text.
(TIF)

S7 Figure **S7 Figure represents identified scaffold material (white pixels) after processing of images retrieved after addition of a contrast agents to the PBS.** These masked binary images are applied in combination with the images with identified tissue (S6 Figure) to establish 3D models from the full stack as presented in Fig. 3 in the main text.
(TIF)

S1 Table **S1 Table represents the data retrieved after post-imaging processing as presented as average in** **Fig. 4B** **.** The first page table results from the analysis of a scan of donor 1 after 5 weeks of culture. Pages 2 and 3 represent the results for donor 2 after 5 weeks of culture. The first column in pink presents the slide identification number of the slide within the complete stack. The second column represents the number of pixels that were identified as tissue. The third column represents the number of pixels identified as scaffold. The pore volume is identified as the number of pixels within the region of interest (ROI) that is not identified as scaffold material. The total number of pixels in the ROI is defined as the number of pixels within a circle that matched the outer ring of the bottom of the scaffold. The percentage of tissue is determined as the number of pixels identified as tissue divided by the number of pixels identified as available pore volume times 100%. The last two columns represent the average tissue amount and the standard deviation on tissue amount.
(PDF)

## References

[1] Jukes JM, Both SK, Leusink A, Sterk LM, van Blitterswijk CA, et al (2008) Endochondral bone tissue engineering using embryonic stem cells. Proc Natl Acad Sci U S A 105:6840–6845. 18467492 10.1073/pnas.0711662105 PMC2374550

[2] Scotti C, Tonnarelli B, Papadimitropoulos A, Scherberich A, Schaeren S, et al (2010) Recapitulation of endochondral bone formation using human adult mesenchymal stem cells as a paradigm for developmental engineering. Proc Natl Acad Sci U S A 107:7251–7256. 20406908 10.1073/pnas.1000302107 PMC2867676

[3] Baradez MO, Marshall D (2011) The use of multidimensional image-based analysis to accurately monitor cell growth in 3D bioreactor culture. PLoS One 6:e26104. 22028809 10.1371/journal.pone.0026104 PMC3197601

[4] Chen WL, Huang CH, Chiou LL, Chen TH, Huang YY, et al (2010) Multiphoton imaging and quantitative analysis of collagen production by chondrogenic human mesenchymal stem cells cultured in chitosan scaffold. Tissue Eng Part C Methods 16:913–920. 19908965 10.1089/ten.TEC.2009.0596

[5] Washburn NR, Weir M, Anderson P, Potter K (2004) Bone formation in polymeric scaffolds evaluated by proton magnetic resonance microscopy and X-ray microtomography. Journal of Biomedical Materials Research Part A 69A:738–747. 10.1002/jbm.a.30054 15162416

[6] Barbetta A, Bedini R, Pecci R, Dentini M (2012) Role of X-ray microtomography in tissue engineering. Annali Dell Istituto Superiore Di Sanita 48:10–18. 22456010 10.4415/ANN_12_01_03

[7] Kofidis T, Lenz A, Boublik J, Akhyari P, Wachsmann B, et al (2003) Pulsatile perfusion and cardiomyocyte viability in a solid three-dimensional matrix. Biomaterials 24:5009–5014. 14559014 10.1016/s0142-9612(03)00429-0

[8] Xu HH, Othman SF, Magin RL (2008) Monitoring Tissue Engineering Using Magnetic Resonance Imaging. Journal of Bioscience and Bioengineering 106:515–527. 19134545 10.1263/jbb.106.515

[9] Appel AA, Anastasio MA, Larson JC, Brey EM (2013) Imaging challenges in biomaterials and tissue engineering. Biomaterials 34:6615–6630. 23768903 10.1016/j.biomaterials.2013.05.033 PMC3799904

[10] Nissi MJ, Rieppo J, Toyras J, Laasanen MS, Kiviranta I, et al (2006) T(2) relaxation time mapping reveals age- and species-related diversity of collagen network architecture in articular cartilage. Osteoarthritis Cartilage 14:1265–1271. 16843689 10.1016/j.joca.2006.06.002

[11] Xia Y, Moody JB, Burton-Wurster N, Lust G (2001) Quantitative in situ correlation between microscopic MRI and polarized light microscopy studies of articular cartilage. Osteoarthritis Cartilage 9:393–406. 11467887 10.1053/joca.2000.0405

[12] Mountain KM, Bjarnason TA, Dunn JF, Matyas JR (2011) The functional microstructure of tendon collagen revealed by high-field MRI. Magn Reson Med 66:520–527. 21674618 10.1002/mrm.23036

[13] Ramaswamy S, Greco JB, Uluer MC, Zhang ZJ, Zhang ZL, et al (2009) Magnetic Resonance Imaging of Chondrocytes Labeled with Superparamagnetic Iron Oxide Nanoparticles in Tissue-Engineered Cartilage. Tissue Engineering Part A 15:3899–3910. 19788362 10.1089/ten.tea.2008.0677 PMC2792067

[14] Bhirde A, Xie J, Swierczewska M, Chen X (2011) Nanoparticles for cell labeling. Nanoscale 3:142–153. 20938522 10.1039/c0nr00493f PMC6454877

[15] Hachani R, Lowdell M, Birchall M, Thanh NTK (2013) Tracking stem cells in tissue-engineered organs using magnetic nanoparticles. Nanoscale 5:11362–11373. 24108444 10.1039/c3nr03861k

[16] Yang CY, Tai MF, Chen ST, Wang YT, Chen YF, et al**.** (2009) Labeling of human mesenchymal stem cell: Comparison between paramagnetic and superparamagnetic agents. Journal of Applied Physics 105 : -.

[17] Chesnick IE, Mason JT, Giuseppetti AA, Eidelman N, Potter K (2008) Magnetic resonance microscopy of collagen mineralization. Biophysical Journal 95:2017–2026. 18487295 10.1529/biophysj.107.120923 PMC2483767

[18] de Bruijn JD, van den Brink I, Mendes S, Dekker R, Bovell YP, et al (1999) Bone induction by implants coated with cultured osteogenic bone marrow cells. Adv Dent Res 13:74–81. 11276750 10.1177/08959374990130011801

[19] Moroni L, Hendriks JA, Schotel R, de Wijn JR, van Blitterswijk CA (2007) Design of biphasic polymeric 3-dimensional fiber deposited scaffolds for cartilage tissue engineering applications. Tissue Eng 13:361–371. 17504063 10.1089/ten.2006.0127

[20] Beumer GJ, van Blitterswijk CA, Ponec M (1994) Biocompatibility of a biodegradable matrix used as a skin substitute: an in vivo evaluation. J Biomed Mater Res 28:545–552. 8027095 10.1002/jbm.820280504

[21] Moroni L, de Wijn JR, van Blitterswijk CA (2005) Three-dimensional fiber-deposited PEOT/PBT copolymer scaffolds for tissue engineering: influence of porosity, molecular network mesh size, and swelling in aqueous media on dynamic mechanical properties. J Biomed Mater Res A 75:957–965. 16118789 10.1002/jbm.a.30499

[22] Leferink AM, Hendrikson WJ, Rouwkema J, Karperien M, van Blitterswijk CA, et al**.** (2013) Increased cell seeding efficiency in bioplotted three-dimensional PEOT/PBT scaffolds. Journal of Tissue Engineering and Regenerative Medicine: n/a-n/a. 10.1002/term.1842 24668928

[23] Cengelli F, Maysinger D, Tschudi-Monnet F, Montet X, Corot C, et al (2006) Interaction of functionalized superparamagnetic iron oxide nanoparticles with brain structures. Journal of Pharmacology and Experimental Therapeutics 318:108–116. 16608917 10.1124/jpet.106.101915

[24] Grootendorst DJ, Jose J, Fratila RM, Visscher M, Velders AH, et al (2013) Evaluation of superparamagnetic iron oxide nanoparticles (Endorem (R)) as a photoacoustic contrast agent for intra-operative nodal staging. Contrast Media & Molecular Imaging 8:83–91. 23109396 10.1002/cmmi.1498

[25] Schindelin J, Arganda-Carreras I, Frise E, Kaynig V, Longair M, et al (2012) Fiji: an open-source platform for biological-image analysis. Nature Methods 9:676–682. 22743772 10.1038/nmeth.2019 PMC3855844

[26] Gordillo N, Montseny E, Sobrevilla P (2013) State of the art survey on MRI brain tumor segmentation. Magnetic Resonance Imaging 31:1426–1438. 23790354 10.1016/j.mri.2013.05.002

[27] Lorensen WE, Cline HE (1987) Marching cubes: A high resolution 3D surface construction algorithm. ACM SIGGRAPH Computer Graphics 21:163–169.

[28] Doube M, Klosowski MM, Arganda-Carreras I, Cordelieres FP, Dougherty RP, et al (2010) BoneJ Free and extensible bone image analysis in ImageJ. Bone 47:1076–1079. 20817052 10.1016/j.bone.2010.08.023 PMC3193171

[29] Taubin G (1995) A signal processing approach to fair surface design. ACM. pp. 351–358.

[30] Abarrategi A, Fernandez-Valle ME, Desmet T, Castejon D, Civantos A, et al (2012) Label-free magnetic resonance imaging to locate live cells in three-dimensional porous scaffolds. J R Soc Interface 9:2321–2331. 22442095 10.1098/rsif.2012.0068 PMC3405747

[31] Constantinidis I, Sambanis A (1998) Noninvasive monitoring of tissue-engineered constructs by nuclear magnetic resonance methodologies. Tissue Engineering 4:9–17. 10.1089/ten.2005.11.404 15869419

[32] Kotecha M, Klatt D, Magin RL (2013) Monitoring cartilage tissue engineering using magnetic resonance spectroscopy, imaging, and elastography. Tissue Eng Part B Rev 19:470–484. 23574498 10.1089/ten.teb.2012.0755 PMC3826474

[33] Neves AA, Medcalf N, Smith M, Brindle KM (2006) Evaluation of engineered meniscal cartilage constructs based on different scaffold geometries using magnetic resonance imaging and spectroscopy. Tissue Engineering 12:53–62. 16499442 10.1089/ten.2006.12.53

[34] Peptan IA, Hong L, Xu HH, Magin RL (2006) MR assessment of osteogenic differentiation in tissue-engineered constructs. Tissue Engineering 12:843–851. 16674297 10.1089/ten.2006.12.843

[35] Burg KJ, Delnomdedieu M, Beiler RJ, Culberson CR, Greene KG, et al (2002) Application of magnetic resonance microscopy to tissue engineering: a polylactide model. J Biomed Mater Res 61:380–390. 12115463 10.1002/jbm.10146

[36] Belaroussi B, Milles J, Carme S, Zhu YM, Benoit-Cattin H (2006) Intensity non-uniformity correction in MRI: existing methods and their validation. Med Image Anal 10:234–246. 16307900 10.1016/j.media.2005.09.004

[37] Milles J, Zhu YM, Gimenez G, Guttmann CR, Magnin IE (2007) MRI intensity nonuniformity correction using simultaneously spatial and gray-level histogram information. Comput Med Imaging Graph 31:81–90. 17196790 10.1016/j.compmedimag.2006.11.001

[38] Fishbein KW, Canuto HC, Bajaj P, Camacho NP, Spencer RG (2007) Optimal methods for the preservation of cartilage samples in MRI and correlative biochemical studies. Magn Reson Med 57:866–873. 17457874 10.1002/mrm.21189

[39] Zheng S, Xia Y (2010) Changes in Proton Dynamics in Articular Cartilage Caused by Phosphate Salts and Fixation Solutions. Cartilage 1:55–64. 21379397 10.1177/1947603509359784 PMC3048334

[40] Crowe JJ, Grant SC, Logan TM, Ma T (2011) A magnetic resonance-compatible perfusion bioreactor system for three-dimensional human mesenchymal stem cell construct development. Chemical Engineering Science 66:4138–4147.

