# Supplementary figures and images for: An Open Source Image Processing Method to Quantitatively Assess Tissue Growth after Non-Invasive Magnetic Resonance Imaging in Human Bone Marrow Stromal Cell Seeded 3D Polymeric Scaffolds
Source: PLoS One. 2014 Dec 12;9(12):e115000. doi: 10.1371/journal.pone.0115000 (PMC4264848; doi:10.1371/journal.pone.0115000)

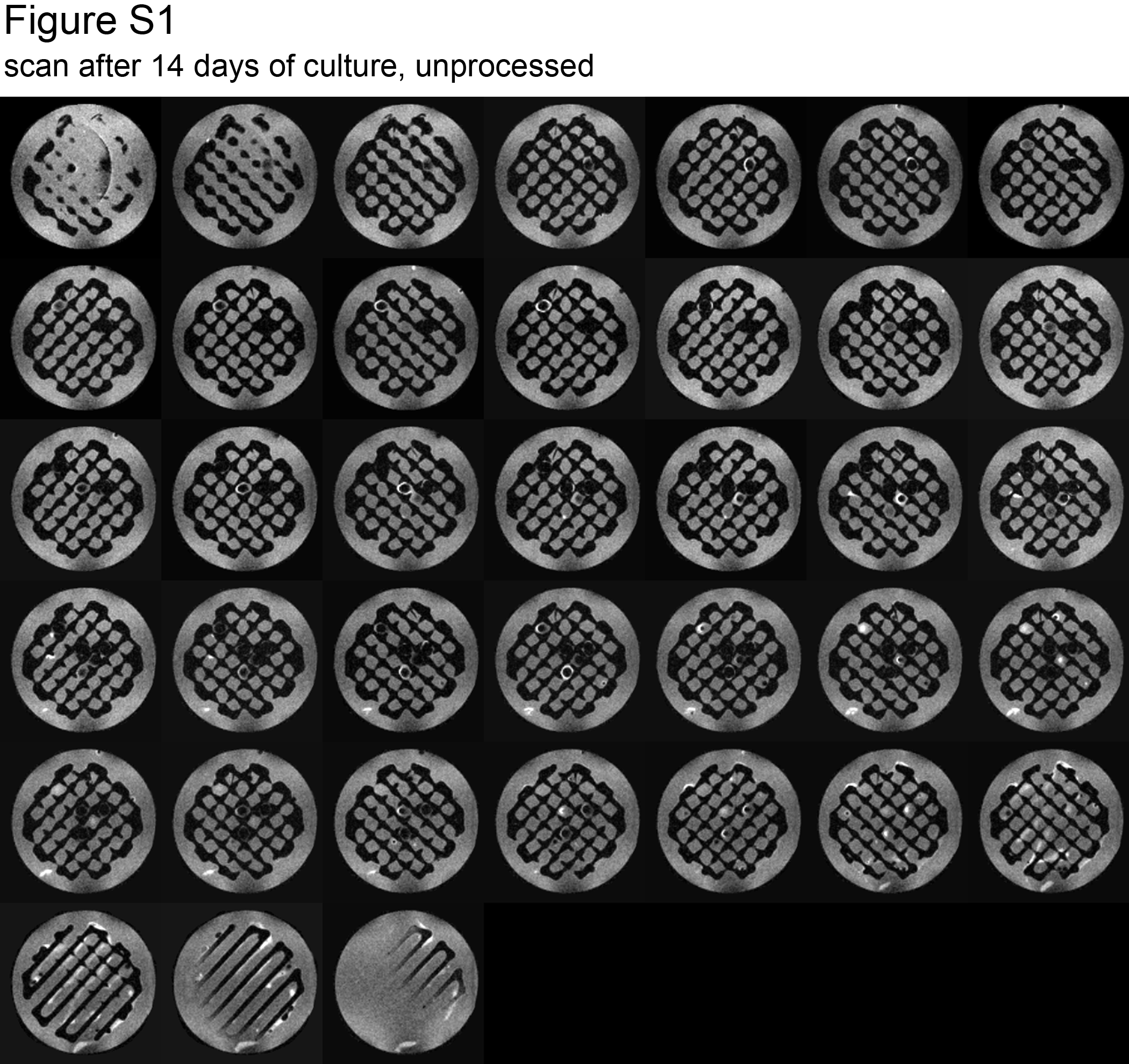

Supplement: S1 Figure — represents the unprocessed images of a scan presented in Fig. 1 in the main text. The scan was performed after 14 days of culture. The last few slides of the stack show higher amounts of white areas which could represent tissue-like material. (TIF) [file pone.0115000.s001.tif]

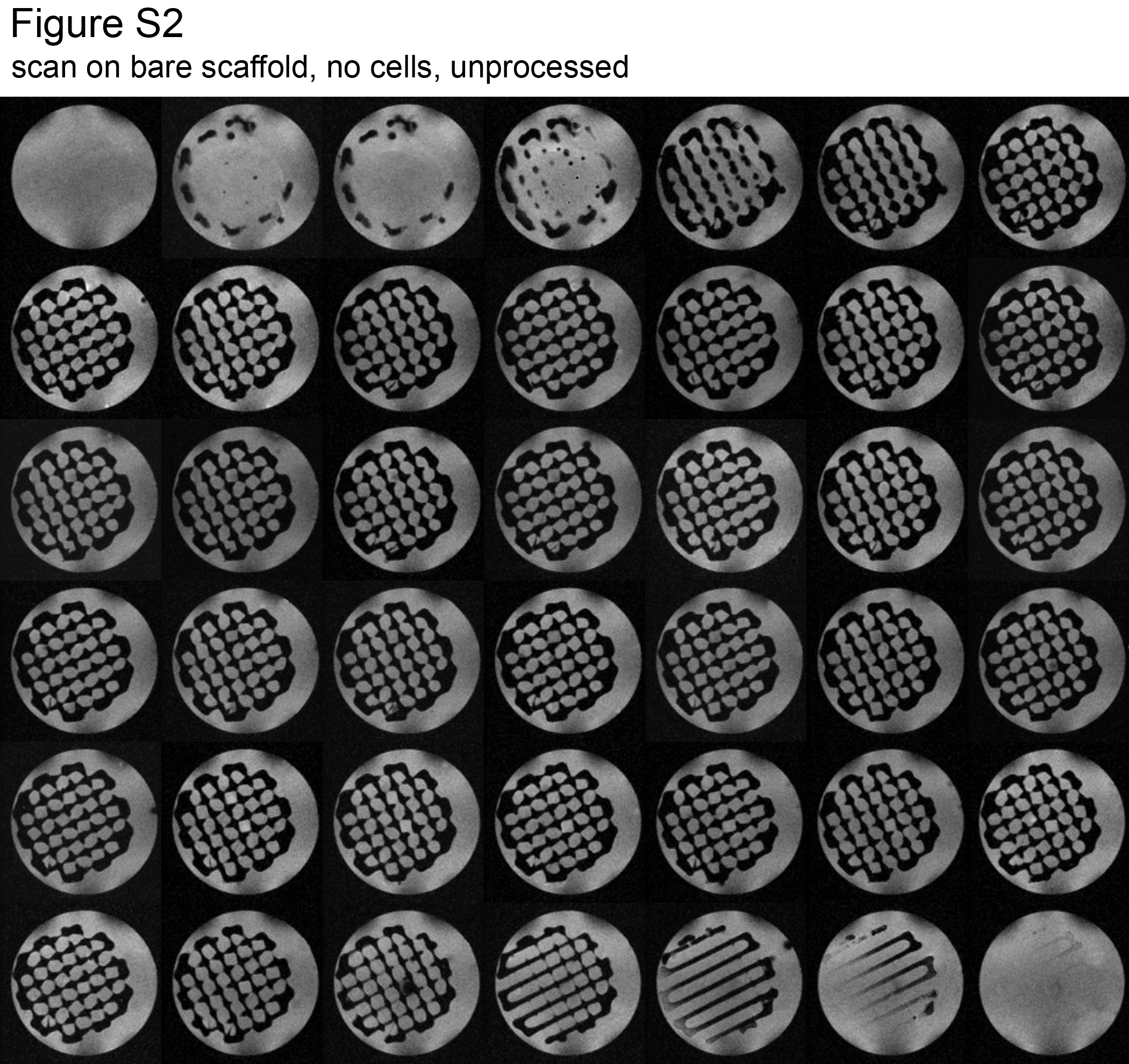

Supplement: S2 Figure — represents the unprocessed images of a scan presented in Fig. 2 in the main text. The scan was performed on a bare scaffold without any cells. There are no evident white areas with similar patterns as found on scans of scaffolds cultured with cells. The scan of the bare scaffold shows strong contrast between the scaffold material and the surrounding PBS. (TIF) [file pone.0115000.s002.tif]

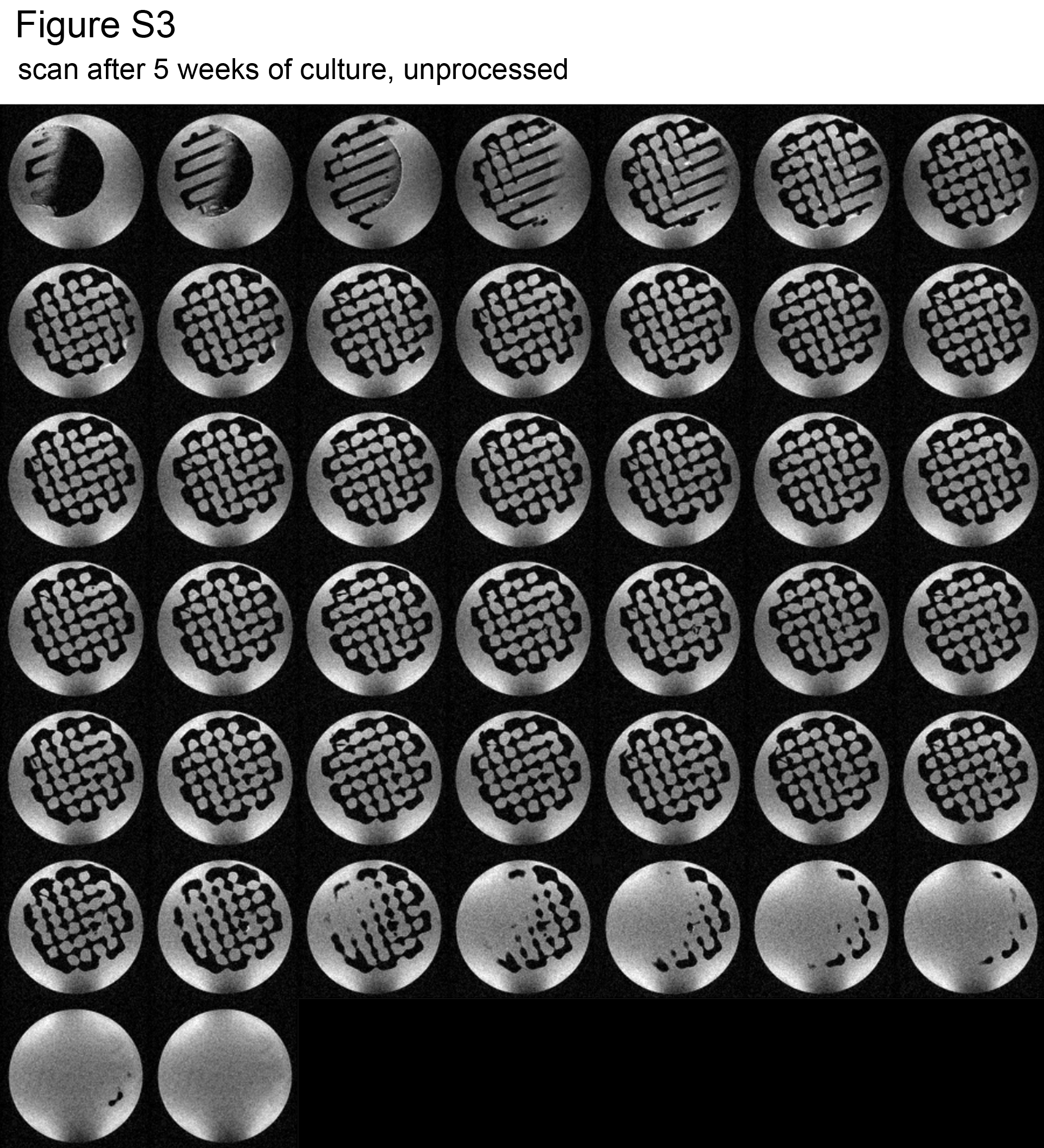

Supplement: S3 Figure — represents the unprocessed images of a scan performed on the same scaffold as S2 Figure yet after 5 weeks of culture. There are small white lines visible in the first few slices of the stack. These white lines show similar patterns as found on other scans of scaffolds cultured with cells. (TIF) [file pone.0115000.s003.tif]

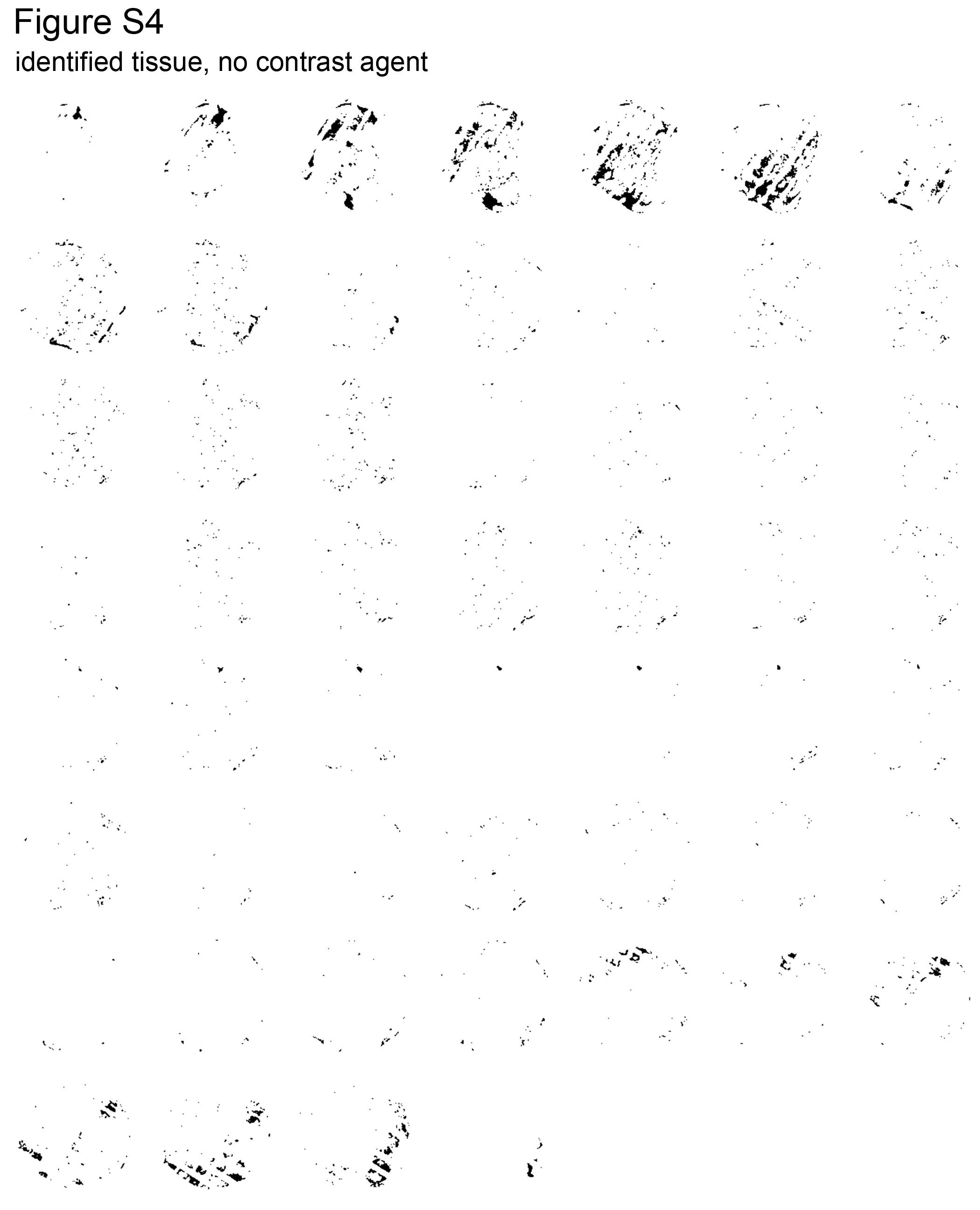

Supplement: S4 Figure — represents identified tissue-like material (black pixels) after processing of images retrieved without the application of contrast agents. These masked binary images are applied to establish 3D models from the full stack as presented in Fig. 3 in the main text. (TIF) [file pone.0115000.s004.tif]

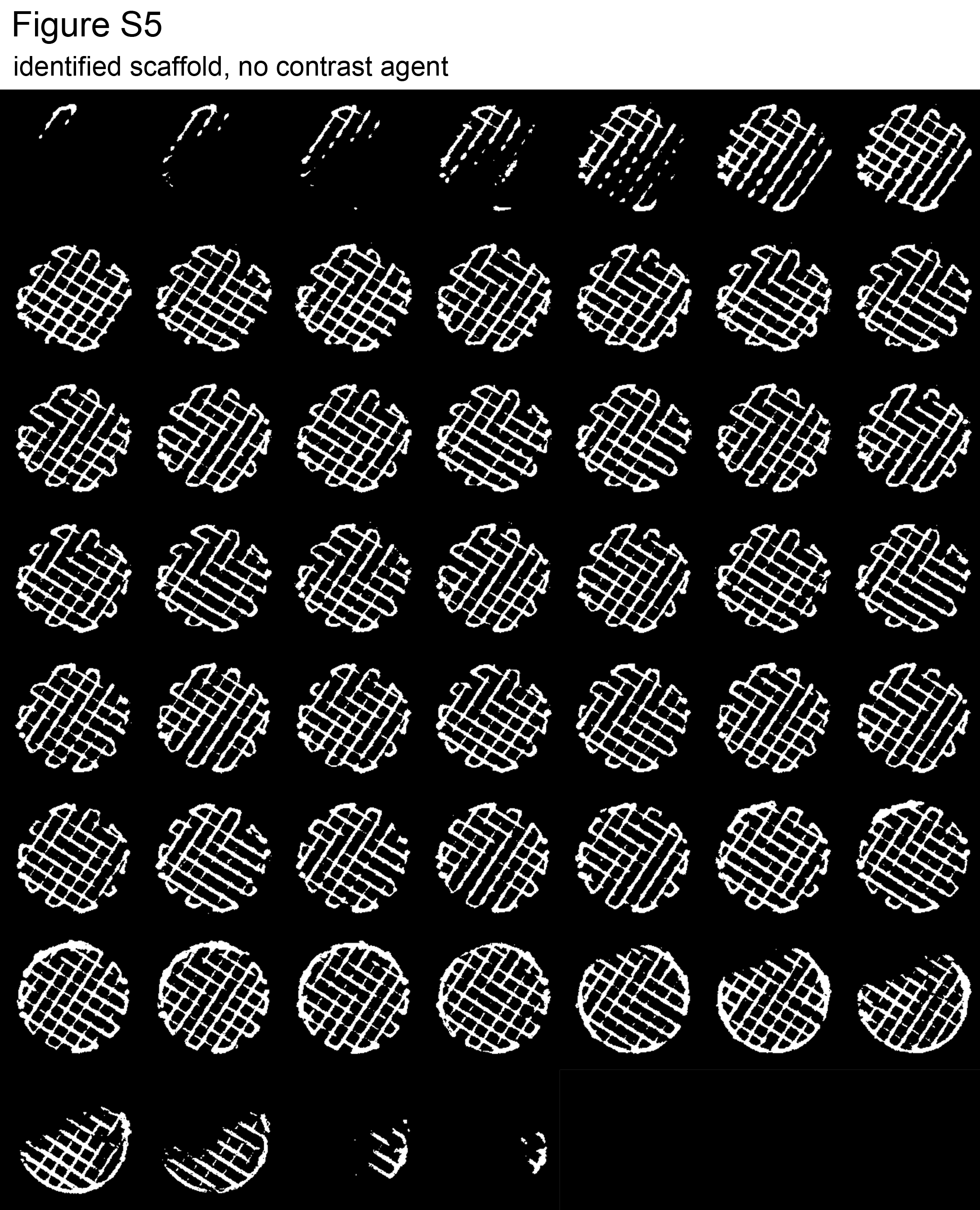

Supplement: S5 Figure — represents identified scaffold material (white pixels) after processing of images retrieved without the application of contrast agents. These masked binary images are applied in combination with the images with identified tissue (S4 Figure) to establish 3D models from the full stack as presented in Fig. 3 in the main text. (TIF) [file pone.0115000.s005.tif]

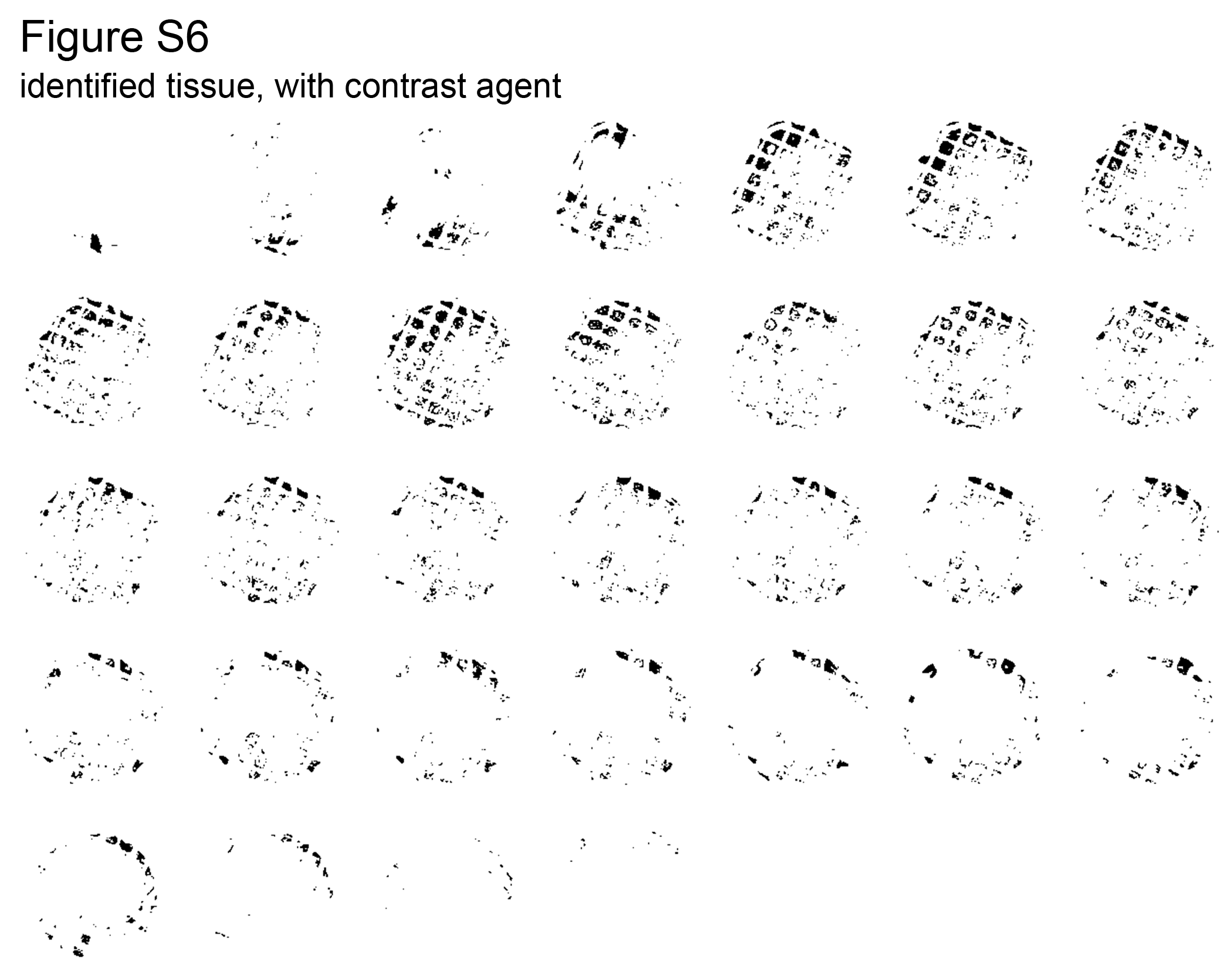

Supplement: S6 Figure — represents identified tissue-like material (black pixels) after processing of images retrieved after addition of a contrast agent to the PBS. These masked binary images are applied to establish 3D models from the full stack as presented in Fig. 3 in the main text. (TIF) [file pone.0115000.s006.tif]

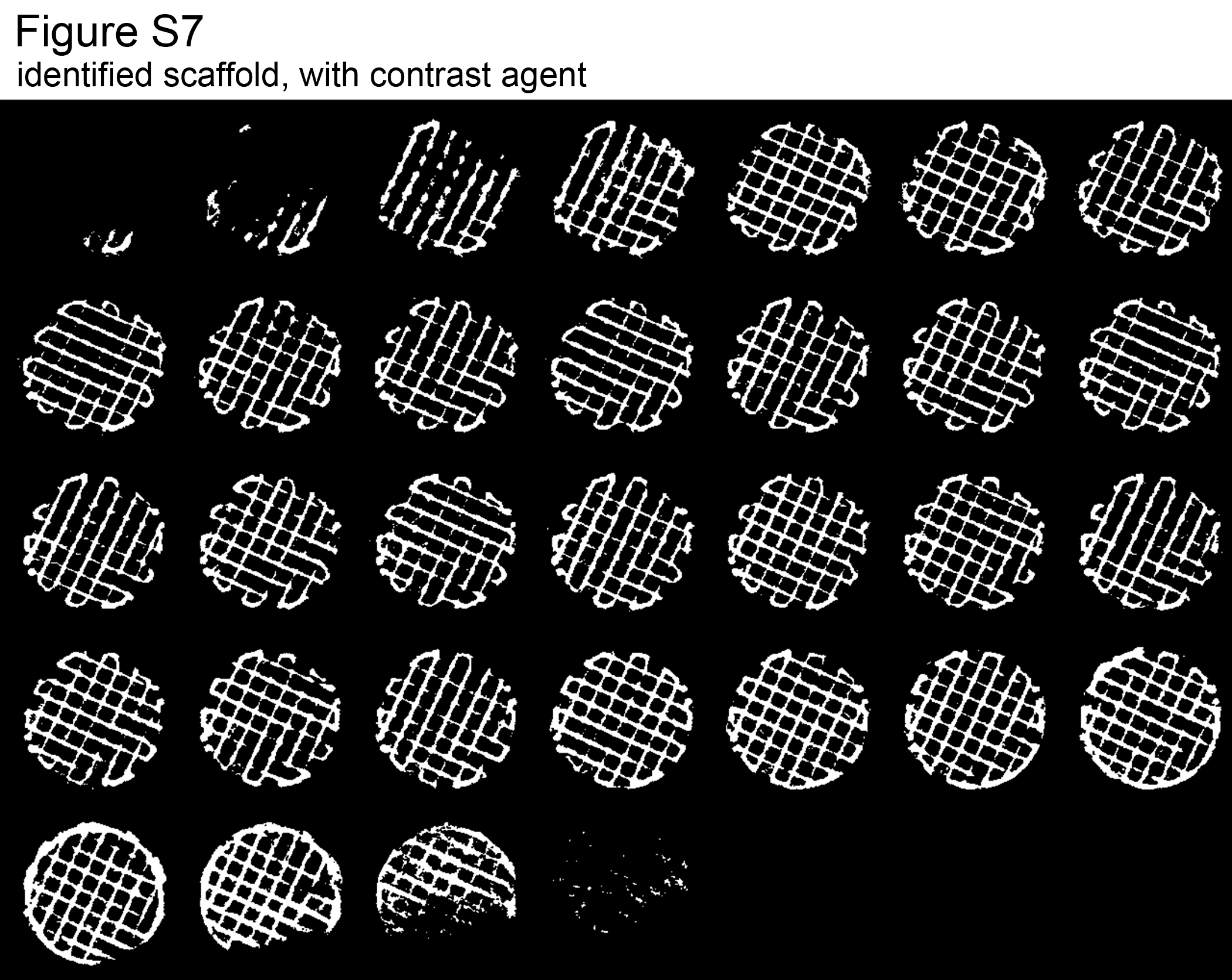

Supplement: S7 Figure — represents identified scaffold material (white pixels) after processing of images retrieved after addition of a contrast agents to the PBS. These masked binary images are applied in combination with the images with identified tissue (S6 Figure) to establish 3D models from the full stack as presented in Fig. 3 in the main text. (TIF) [file pone.0115000.s007.tif]
