# Supplementary material for: An Open Source Image Processing Method to Quantitatively Assess Tissue Growth after Non-Invasive Magnetic Resonance Imaging in Human Bone Marrow Stromal Cell Seeded 3D Polymeric Scaffolds
Source: PLoS One. 2014 Dec 12;9(12):e115000. doi: 10.1371/journal.pone.0115000 (PMC4264848; doi:10.1371/journal.pone.0115000)
Supplement: S1 Table — represents the data retrieved after post-imaging processing as presented as average in Fig. 4B . The first page table results from the analysis of a scan of donor 1 after 5 weeks of culture. Pages 2 and 3 represent the results for donor 2 after 5 weeks of culture. The first column in pink presents the slide identification number of the slide within the complete stack. The second column represents the number of pixels that were identified as tissue. The third column represents the number of pixels identified as scaffold. The pore volume is identified as the number of pixels within the region of interest (ROI) that is not identified as scaffold material. The total number of pixels in the ROI is defined as the number of pixels within a circle that matched the outer ring of the bottom of the scaffold. The percentage of tissue is determined as the number of pixels identified as tissue divided by the number of pixels identified as available pore volume times 100%. The last two columns represent the average tissue amount and the standard deviation on tissue amount. (PDF) [file pone.0115000.s008.pdf]

MRI005						
DONOR 1			full image nr pixels		537289	bottom
5 weeks	per slice	**per slice**	ROI number of pixels		232868	middle
	tissue	scaffold	pore volume	tissue %		top
RF_AL_ex-bare_5weeks_1	1508	9292	223576	0,67	1,86	1,36
RF_AL_ex-bare_5weeks_2	1627	28167	204701	0,79		
RF_AL_ex-bare_5weeks_3	4114	51138	181730	2,26		
RF_AL_ex-bare_5weeks_4	5787	67194	165674	3,49		
RF_AL_ex-bare_5weeks_5	6517	82937	149931	4,35		
RF_AL_ex-bare_5weeks_6	5879	90720	142148	4,14		
RF_AL_ex-bare_5weeks_7	766	98677	134191	0,57		
RF_AL_ex-bare_5weeks_8	1661	100092	132776	1,25		
RF_AL_ex-bare_5weeks_9	2392	101879	130989	1,83		
RF_AL_ex-bare_5weeks_10	2505	102422	130446	1,92		
RF_AL_ex-bare_5weeks_11	428	105293	127575	0,34		
RF_AL_ex-bare_5weeks_12	3434	105462	127406	2,70		
RF_AL_ex-bare_5weeks_13	550	108792	124076	0,44		
RF_AL_ex-bare_5weeks_14	1597	108586	124282	1,28		
RF_AL_ex-bare_5weeks_15	3171	109012	123856	2,56	1,17	1,09
RF_AL_ex-bare_5weeks_16	1245	110343	122525	1,02		
RF_AL_ex-bare_5weeks_17	4356	108677	124191	3,51		
RF_AL_ex-bare_5weeks_18	2176	109208	123660	1,76		
RF_AL_ex-bare_5weeks_19	142	112132	120736	0,12		
RF_AL_ex-bare_5weeks_20	1248	109889	122979	1,01		
RF_AL_ex-bare_5weeks_21	1545	109508	123360	1,25		
RF_AL_ex-bare_5weeks_22	431	110184	122684	0,35		
RF_AL_ex-bare_5weeks_23	1394	109669	123199	1,13		
RF_AL_ex-bare_5weeks_24	3372	107499	125369	2,69		
RF_AL_ex-bare_5weeks_25	588	108032	124836	0,47		
RF_AL_ex-bare_5weeks_26	500	106899	125969	0,40		
RF_AL_ex-bare_5weeks_27	12	108235	124633	0,01		
RF_AL_ex-bare_5weeks_28	132	106983	125885	0,10		
RF_AL_ex-bare_5weeks_29	406	107514	125354	0,32	0,11	0,17
RF_AL_ex-bare_5weeks_30	731	106436	126432	0,58		
RF_AL_ex-bare_5weeks_31	122	106973	125895	0,10		
RF_AL_ex-bare_5weeks_32	0	109057	123811	0,00		
RF_AL_ex-bare_5weeks_33	71	107683	125185	0,06		
RF_AL_ex-bare_5weeks_34	4	110777	122091	0,00		
RF_AL_ex-bare_5weeks_35	203	110476	122392	0,17		
RF_AL_ex-bare_5weeks_36	41	109517	123351	0,03		
RF_AL_ex-bare_5weeks_37	396	97065	135803	0,29		
RF_AL_ex-bare_5weeks_38	4	73937	158931	0,00		
RF_AL_ex-bare_5weeks_39	51	51895	180973	0,03		
RF_AL_ex-bare_5weeks_40	94	33767	199101	0,05		
RF_AL_ex-bare_5weeks_41	31	19563	213305	0,01		
RF_AL_ex-bare_5weeks_42	8	7022	225846	0,00		
RF_AL_ex-bare_5weeks_43	0	1710	231158	0,00		

MRI run 25 no CA	pixel number by histogram analysis->list->255 after segmentation					
DONOR 2			full image nr pixels		537289	top
5 weeks	per slice	**per slice**	ROI number of pixels		266000	middle
	tissue	scaffold	pore volume	tissue %		bottom
RF_AL_run256w_noCA_6	2.450	5342	260658	0,94	3,61	4,04
RF_AL_run256w_noCA_7	8.274	9491	256509	3,23		
RF_AL_run256w_noCA_8	18.191	15979	250021	7,28		
RF_AL_run256w_noCA_9	19.713	28480	237520	8,30		
RF_AL_run256w_noCA_10	25.169	48840	217160	11,59		
RF_AL_run256w_noCA_11	26.946	70416	195584	13,78		
RF_AL_run256w_noCA_12	6.651	85279	180721	3,68		
RF_AL_run256w_noCA_13	8.223	90197	175803	4,68		
RF_AL_run256w_noCA_14	5.533	84763	181237	3,05		
RF_AL_run256w_noCA_15	1.609	93905	172095	0,93		
RF_AL_run256w_noCA_16	1.358	85457	180543	0,75		
RF_AL_run256w_noCA_17	404	88010	177990	0,23		
RF_AL_run256w_noCA_18	1.814	88878	177122	1,02		
RF_AL_run256w_noCA_19	1.853	80612	185388	1,00		
RF_AL_run256w_noCA_20	2.552	82647	183353	1,39		
RF_AL_run256w_noCA_21	2.105	85869	180131	1,17		
RF_AL_run256w_noCA_22	2.893	86967	179033	1,62		
RF_AL_run256w_noCA_23	467	85689	180311	0,26		
RF_AL_run256w_noCA_24	885	86890	179110	0,49		
RF_AL_run256w_noCA_25	665	82165	183835	0,36	0,60	0,26
RF_AL_run256w_noCA_26	822	83186	182814	0,45		
RF_AL_run256w_noCA_27	740	81903	184097	0,40		
RF_AL_run256w_noCA_28	1.462	76548	189452	0,77		
RF_AL_run256w_noCA_29	1.489	80770	185230	0,80		
RF_AL_run256w_noCA_30	1.857	83613	182387	1,02		
RF_AL_run256w_noCA_31	2.187	81240	184760	1,18		
RF_AL_run256w_noCA_32	993	83719	182281	0,54		
RF_AL_run256w_noCA_33	1.354	80274	185726	0,73		
RF_AL_run256w_noCA_34	771	81621	184379	0,42		
RF_AL_run256w_noCA_35	1.406	83863	182137	0,77		
RF_AL_run256w_noCA_36	1.094	85014	180986	0,60		
RF_AL_run256w_noCA_37	400	85616	180384	0,22		
RF_AL_run256w_noCA_38	461	81092	184908	0,25		
RF_AL_run256w_noCA_39	987	85427	180573	0,55		
RF_AL_run256w_noCA_40	844	83044	182956	0,46		
RF_AL_run256w_noCA_41	1.545	83296	182704	0,85		
RF_AL_run256w_noCA_42	289	88142	177858	0,16		
RF_AL_run256w_noCA_43	361	82966	183034	0,20	1,64	1,84
RF_AL_run256w_noCA_44	1.249	85453	180547	0,69		
RF_AL_run256w_noCA_45	1.028	87483	178517	0,58		
RF_AL_run256w_noCA_46	414	99688	166312	0,25		
RF_AL_run256w_noCA_47	956	93120	172880	0,55		
RF_AL_run256w_noCA_48	480	104750	161250	0,30		
RF_AL_run256w_noCA_49	733	105491	160509	0,46		
RF_AL_run256w_noCA_50	1.471	103620	162380	0,91		
RF_AL_run256w_noCA_51	1.476	101718	164282	0,90		

RF_AL_run256w_noCA_52	4.858	98460	167540	2,90
RF_AL_run256w_noCA_53	2.446	100254	165746	1,48
RF_AL_run256w_noCA_54	5.443	82968	183032	2,97
RF_AL_run256w_noCA_55	8.103	70148	195852	4,14
RF_AL_run256w_noCA_56	14.090	45727	220273	6,40
RF_AL_run256w_noCA_57	10.880	15101	250899	4,34
RF_AL_run256w_noCA_58	1.901	7129	258871	0,73
